# Pennsylvania State Dental Society

**Published:** 1888-09

**Authors:** 


### PENNSYLVANIA STATE DENTAL SOCIETY.

### TWENTIETH ANNUAL MEETING HELD IN PHILADELPHIA, JUNE 5, 6, 7, 8, 1888.

ESPECIALLY REPORTED FOR THE INDEPENDENT PRACTITIONER.

### THURSDAY MORNING SESSION.

DISCUSSION ON DR. TRUMAN'S PAPER CONTINUED.

*Dr. H. C. Register,* of Philadelphia—The paper has delighted me very much indeed. Dr. Truman is a very conservative teacher, consequently a good one. There can be no doubt but that other filling materials save teeth as well as gold. We see this in our practice daily, and the question naturally arises as to the forces at work to bring about the same result even when materials diametrically opposed are used. To my mind the success lies in the knowledge and skill used in preparing the cavity and choosing the material to be inserted. We should not operate upon teeth as we would upon bricks or stone. Instead of an inanimate object, we have to operate upon a highly vitalized one, that is subject to law as much as any other portion of the body. Our operations must be in accordance with the laws which govern the changes that take place if we wish success. The tooth is not a solid organ, but a most composite one. Let us, for a minute, consider the development and histology of a normal tooth. There is a central cavity, surrounded by dentine, containing pulp, nerves and vessels. Upon the surface of the pulp is the odontoblastic layer which served to develop the dentine. The latter is composed of tubuli and basis-substance. The fibrillæ take up the matter from the odontoblasts and pass it into the basis-substance. The amount of organic to inorganic material varies in an adverse ratio to the age of the tooth. It is also a well-known fact that mature teeth also vary very greatly in their density. Many operators in preparing a cavity do not take into consideration the age of the teeth or the density of the tooth to be operated upon. We are too apt to get into a groove in practice, to adopt one method and follow it regardless of the physiological conditions which are known to exist. The teeth of young persons are treated the same as those of persons thirty-five or forty years old. The main thought of some operators seems to be to make a cavity that will hold the filling, whereas, in my judgment, that should be a secondary consideration. Of course we desire to have our fillings remain in, but in order to accomplish this we must take into consideration physiological as well as mechanical laws. No deep undercuts should be formed, but the cavity should be made slightly wedge-shaped. Where deep undercuts are made, the dentinal tubuli are cut through and the vitality of the distal ends destroyed. The devitalized portion of the dentine becomes friable and is easily broken down. Frail margins arise from disregarding physiological as well as mechanical laws. Regarding filling materials, it is a well-known fact that decay does not occur under gutta-percha or cement fillings. It seems that the protection of the dentine is the secret of success in filling teeth. Soft foil should be used in the bottom of the cavity in juxtaposition with the dentine, but harder foil may be used to finish where it comes in contact with the enamel. I believe that where there is perfect adaptation between filling and tooth substance that calcification of the ends of the fibrillæ occurs, and that the surface of the dentine is better prepared to withstand decay.

In forming cavities I cut down from the crown. I do not use retaining pits. I form my cavity so that the interior is slightly larger than the outer portion, then by use of a matrix I work soft-like plastic material, finishing with cohesive foil, using a small retaining pit in the crown if necessary. A surface hardness sufficient for all purposes is thus obtained. All pure foils are cohesive, and those that are non-cohesive are made so by some addition to their composition. Almost all foils that are sold for non-cohesive can be made cohesive by heating, but they are apt to discolor. There are certain foils, however, that contain alloy which cannot be so treated. Those that can be made cohesive have received only a coating upon the surface, and which is evaporated by the heat.

*Dr. Kirk*—The matter I want to speak of has been gone over largely by the gentlemen who have preceded me. I wish, however, to emphasize one or two points brought out, as they are of special interest. The terms "hard and cohesive," "soft and non-cohesive," as applied to dental gold foils, have been used as though they were perfectly interchangeable. Such use of the terms, however, is erroneous. Pure gold is inherently soft and inherently cohesive, and deviation from these conditions is the result of impurity in the foil or upon its surface. I have carefully investigated the matter myself, and have had numbers of assays made of the principal foils in the market, the results of which work all tend to prove the correctness of this conclusion. There is no variation from this law of stability in any department of nature. For example, pure water has certain physical attributes which are fixed and invariable, such as transparency, a fixed boiling point, specific gravity, etc., which are always the same under like conditions. This same thing is true of every other element or definite compound in nature, and when we have books of dental gold foil sent us from the manufacturers, bearing on the outside statements to the effect that "this foil is absolutely pure," and then is denominated cohesive, semi-cohesive, non-cohesive, or soft, as the case may be, it certainly appears incongruous.

I had occasion while writing the metallurgical article in the American System of Dentistry, to endeavor, if possible, to ascertain the cause of the differences observed in the physical properties of dental gold foils. I purchased books of different makes of gold foil in the open market, and had them tested by the assayer of the Philadelphia mint (than whom no higher authority on this subject exists), and the results obtained were, to say the least, interesting. I found in the soft foil of Abbey, which is claimed to be absolutely pure, nearly two parts in a thousand of impurity; whether this impurity was an integral part of the foil I do not know. I do not, however, believe, like Dr. Register, that it was a surface impurity. Certainly it was not volatile, as the foil was subjected to a red heat for several minutes before the assay was made, in order that all volatile matters should be driven off. All the other foils examined were subjected to the same treatment, viz.: heating to redness before the assays were made. Of all those examined, Abbey's foil was found to be the most impure from a chemical standpoint. I found the foil made by Rowan, of Jersey City, to approach the nearest to absolute purity. This latter foil, and that of the S. S. White Dental Manufacturing Co., known as "Quarter Century" foil, contained the least impurity of any—a mere fraction of a part in a thousand. These two foils, when manipulated under similar conditions, were almost indistinguishable from each other; but between these foils and that of Abbey's there was a wide difference physically. I do not take the position that the impurity found in Abbey's foil was a detriment to it, nor would the ultra refinement of the Rowan and White foils, on the other hand, necessarily give them a special superiority for the methods of manipulation. The two classes of foils are so entirely different, and depend so largely upon distinctive properties which they each possess, and which in the one case is dependent upon the presence of some debasing material, that a comparison as to their working value can scarcely be made.

What I wish to bring out is that the difference in physical properties is due, not to any inherent difference in the gold as gold, but to the slight admixture of some other substances which exercise a marked modifying influence upon the physical properties of the finished foil.

The difference in the kind of impurity exercises an influence on the foil fully as great as the difference in the amount which it contains. Thus, if the alloy or impurity is an oxidizable metal, the effect upon the foil is very different in kind and in degree from that produced by a union with one of the noble metals, such as platinum or silver, that are not ordinarily oxidizable. The base metals, such as lead or tin, not only confer hardness upon the foil, but extreme brittleness. Take a foil so contaminated, and anneal it as thoroughly as you please, and still I believe it is beyond the highest dental skill to make a filling with it which will be moisture tight and prevent a recurrence of decay.

As an illustration of the physical properties of absolutely pure gold, I had made at the mint a sample of gold, which the authorities there guaranteed to be absolutely 1,000 fine. I had this gold beaten carefully, and with special precautions, into No. 4 dental foil. I tested it myself in fillings, and also gave portions of it to other practitioners, and they all agreed with me as to its extreme softness and cohesiveness. It was scarcely distinguishable from the Wolrab foil, or any other absolutely pure foil. I took some sheets of it, put them into the book of a foil manufacturer, and had them assayed at the mint, and they told me it was the only sample of the lot of foils which I brought to them for examination which was absolutely pure; they having had no knowledge of the fact that they were making an assay of their own proof gold.

In view of the results which I have sketched, I cannot help feeling that much of the defective gold work that is done is, to a considerable degree, due to the harshness or unworkable character of the foils used. I am also convinced that a filling, or I will use the term "plug" in this instance, can be made absolutely moisture tight, and permanently protect the tooth from a recurrence of decay by the use of absolutely pure gold which contains in itself the greatest softness with a maximum of cohesiveness.

This part of the subject has been well brought out by those who have preceded me, so I only arose to view the matter from its chemical aspect.

*Dr. Ward*—May I ask Dr. Kirk whether the impurities are in the soft foil or others?

*Dr. Kirk*—I had intended to state that the soft foil of Abbey has a reputation, and we all know what it will do. I do not stand here to criticise and make a point against Abbey's foil because of its impurity, because that very impurity which exists in Abbey's foil has an advantage, and I do not believe we could get the results with Abbey's foil if this impurity did not exist, because it adds a certain property to it. Nor do I say that an absolutely pure gold foil is a great desideratum. But we must know that these two words of softness and cohesiveness do belong to pure gold.

AFTERNOON SESSION.

Paper read by Dr. C. N. Peirce, on "The Development and Eruption of the Permanent Teeth."

DISCUSSION.

*Dr. James Truman*—Dr. Peirce has opened a broad subject, and one that has been discussed in its general details, oftentimes, I have thought, without much profit. I cannot agree with him, however, that the jaw is decreasing in size, that it follows necessarily that we must lose a tooth. You must remember that going back to the higher anthropoid apes we find their molars, bicuspids and premolars very much larger than those of the human race, and as we come up further to the cave skulls we find that while they are probably somewhat decreased in size, yet still very much larger than the teeth of an ordinary civilized individual at the present time. As man becomes more civilized, the teeth decrease in size, assuming more and more an equilibrium, consequently I do not think that it is at all necessary that the present human race should lose a single tooth for the purpose of articulation. I regard the sixth-year molar, so-called, as one of the most important teeth in the whole series—broad upon its masticating surface; it is, in fact, the key of the arch, which, if taken out, destroys the symmetry of the arch to a certain extent, and oftentimes so seriously as to impair the occlusion, and possibly mastication. I hold that it is impossible to remove any tooth in the jaw without this following. When you remove the sixth-year molar, you necessarily throw the front teeth back, because they assume the direct vertical, and articulation is likely to be impaired. Why then remove the sixth-year molar to articulate the wisdom or third molar ? Some persons entertain queer ideas regarding the loss of the third molar, simply because they are found misplaced in some individuals. It is, perhaps, only a temporary inheritance, and equilibrium is sure to come in the process of evolution. We will have irregularities during that period, but the time is bound to come when it will be found that all the teeth are necessary, and every one can be retained in its proper condition.

*Dr. Guilford*—As Dr. Truman has said, this is a large subject opened by Dr. Peirce, so that we are liable to take different views of the subject. It is certainly a very important matter. We must first study the etiology of this condition. It is very evident that there is a malposition in the third molars at the present time that did not previously exist. All the different writers who have written upon this subject, and have examined the different collections in the different museums, have failed to show any evidences in regard to the peculiar condition of the third molars to-day. I shall have to disagree with what Dr. Truman has said in regard to the jaws. I think Dr. Peirce is right in regard to the size of the teeth and jaw. I think the human frame is, to a certain extent, decreasing in size. I believe the maxillary bone is smaller, but we have no evidence that the teeth are becoming any smaller. You will probably remember that in the earliest recorded skulls found that the teeth were no larger than our own. In reports of examinations of the skulls in Egypt and Mount Sinai, no mention is made regarding any unusual size of teeth. The reports are conflicting also regarding the teeth of giants. In some cases the teeth are very large, and in others not. As I take it, the size of the teeth has not changed, but the jaws have. One way I come to this conclusion is in the unusual number of cases of irregularities existing at the present day. The question comes back to us, why should teeth erupt in that way ? It is due, I think, to two causes. The first has been mentioned by Dr. Peirce, viz., the decrease in the size of the jaw; the other is due to the third molars coming in at a late age. When they come in at twenty-one or twenty-two years, the processes are soft, and the other teeth are easily moved, but when eruption is retarded until the thirtieth or thirty-fifth year, the processes are then hard, and they come through with great difficulty. I remember a case quite a number of years ago of one of our members. He had a large jaw and large teeth. They were erupted too late, and he was confined to his room for two weeks, and suffered intensely. Was it due to a lack of proportions of the jaw, or too late eruption of the teeth ?

Dr. Peirce spoke of anticipating this difficulty. He suggested that it was possible to foretell, in some cases, when there was evidence that there would be trouble. I do not think our foreknowledge goes that far. If the individual has large teeth and small jaws, we might possibly treat in that way, but until the time comes we do not know how far the process is going to enlarge. If it does not enlarge, then extract the second molar, as I do in my practice.

*Dr. Smith*—The matter of knowing what kind of a patient you are operating on is of vital importance, so far as inflammatory influences are concerned. It is a matter that should be inquired into. We cannot make a correct prognosis of any case without a good grounded history of the subject of the case. That is a law in surgery, and therefore it is better not to risk operations. We ought, of course, to mitigate the sufferings, but it is best not to go ahead without being prepared for the results. Sometimes an operation performed on the subject, without waiting further time, causes destruction of the maxillary bone. The impaction of the wisdom teeth is not, of course, a general thing, and it may be called an anomalous condition. We only find it in persons whose jaws are not perfectly developed. They are isolated cases in surgery, and I am glad to say it is not a general thing in the human family. When we look at the German race we find a development of the superior and inferior maxillary bones that is superior to the American jaws, and we can reflect it back to the manner of living. These bones may be retarded in growth by the food which is taken into the system, and by the manner of life the person leads. We find in the German jaw, and in those who subsist upon the hard black bread, better maxillary bones than those fed upon the delicacies of the

American table. We all know that in the south, among the slaves, larger bones and better jaws were found in those living on plain fare than those nourished in the houses of the planters. With regard to the sixth-year molar I believe no rule can be laid down for it. The only thing to do is to make close observation from the patients themselves, and in regard to whether these children are from scrofulous parentage or not, it should not be a matter of delicacy for the dentist to inquire into this matter. They have as good a right to inquire into the heritage of the family as the physician.

*Dr. Fordham*—I am a great friend of the sixth-year molar, and in my practice the loss of it has given me great annoyance, and great annoyance to my patients. I have in my mind now a physician who, when he came to me, had lost his lower sixth-year molar, and the twelfth-year molar was lying upon its side as if trying to do the work of two. As to myself, personally, I would sooner lose the twelfth-year molar than the sixth-year, for it would then leave an unbroken arch to the twelfth-year. It is well known that the twelfth-year molar is often inferior to the sixth, and unless the latter is so bad that it cannot be saved, I do not extract the sixth, but crown it, and if the twelfth is bad extract it. In these days when we can perform such almost miraculous operations, it seems entirely unnecessary to lose this sixth-year molar, taking into account that the twelfth-year molar is so apt to be inferior.

*Dr. James Truman*—I simply want to add to what I said before, that it seems to me that a great many persons in considering this subject take a narrow view of it. When we take into consideration that all race types—at least so far as my observation goes—are not troubled with the wisdom teeth or the sixth-year molar, but their teeth keep a normal position in the jaw in almost all cases, notably the Europeans, South Sea Islanders, Aborigines of America, etc., then it seems to me that there is a cause for this difficulty. The race here is not a true type, but is approaching one. It may be one hundred years before we reach the true American type. We are a mixed people, coming from all races. We have not reached that equilibrium that I spoke of. The time is, however, bound to come when the American race will assume its own type. It is much better than it was one hundred years back, and will be better one hundred years in the future. I do not see how we can avoid this conclusion. If we will only extend our observation to all the races of the earth, we will not find this difficulty. It is not the proper way to look at the thing. Dr. Guilford says the teeth before were not larger. I think I can show in the caveman in Belgium that the teeth are larger all the way back.

Paper read by Dr. L. Ashley Faught, on "Our Dental Literature."

DISCUSSION.

*Dr. Gerhart*—As a member of the Examining Board for a number of years, it has been my fortune to look upon the writings of a great many men who come up for examination, and it has been to me sometimes a matter of great astonishment. My home is in a university town, where I have good opportunities for observation, and I have almost invariably found that the men who finish their course in that university and afterwards follow their studies in the various professional schools, have been better equipped, not so much for what they have learned there, but because of the discipline that has been received. It is the duty of the faculties of our colleges to demand of matriculates that if they have not a college education they shall have, at least, had an advanced academic course.

*Dr. James Truman*—It is not a subject for discussion, exactly, but I was very much pleased with the paper. I hope it will have a broad distribution. The dental profession needs it. The fact that so few have taken a part in the literary work of our profession is a disgrace to it. Every man is able to do something. We are, perhaps, guilty of taking the work of other men and putting our name to it, and sending it out broadcast. I am glad Dr. Faught has brought the matter up in the way he has.

*Dr. Wm. Trueman*—From the showing just made there seem so few contributors to the dental journals that I can hardly believe it. To be sure, I have noticed that the same names are repeated very often. I have also been surprised in looking over the index of the *Dental Cosmos* to find such a comparatively short list of contributors. Another point brought out was the very few original contributions. It is often hard work and nothing to show for it. We may start a series of experiments and find we have been following up a blind lead, and we must confess that there is a great deal of discouragement in this matter. I do not know how our profession compares with the medical profession. I have sometimes thought that a little more literary talent was shown in their compositions, and that there were more contributors.

*Dr. Smith*—I do not see much difference between the dental and medical profession in this respect. I do not think the dental profession is much behind. I am ready to stand up for the dentists. We must remember that the dental is not as old as the medical fraternity. I think dentists have done a great deal within the last decade, at least as far as originality in their productions is concerned, but it must be remembered that writers are not altogether original, and it is clearly impossible to ask of the writer to be altogether so. We could count them on our fingers if we would have only original communications in our literature. I think we can enlarge upon other men's thoughts to good advantage, and that is what we want. We want to develop and interchange ideas. We do not want to depend upon one journal; there are other journals in this State. There are other journals that are giving good literature. We have some of the most able writers in other journals, and why do we stand and criticise dental literature by the productions of one journal. There are leading thoughts from good minds besides those found in the *Dental Cosmos.*

(TO BE CONTINUED.)

